# Structural basis of human PDZD8–Rab7 interaction for the ER-late endosome tethering

**DOI:** 10.1038/s41598-021-98419-5

**Published:** 2021-09-22

**Authors:** Haider Khan, Lin Chen, Lingchen Tan, Young Jun Im

**Affiliations:** College of Pharmacy, Chonnam National University, Gwangju, 61186 Republic of Korea

**Keywords:** X-ray crystallography, Membrane lipids, Intracellular signalling peptides and proteins

## Abstract

The membrane contact sites (MCSs) between the ER and late endosomes (LEs) are essential for the regulation of endosomal protein sorting, dynamics, and motility. PDZD8 is an ER transmembrane protein containing a Synaptotagmin-like Mitochondrial lipid-binding Proteins (SMP) domain. PDZD8 tethers the ER to late endosomes and lysosomes by associating its C-terminal coiled-coil (CC) with the LE Rab7. To identify the structural determinants for the PDZD8–Rab7 interaction, we determined the crystal structure of the human PDZD8 CC domain in complex with the GTP-bound form of Rab7. The PDZD8 CC contains one short helix and the two helices forming an antiparallel coiled-coil. Two Rab7 molecules bind to the opposite sides of the PDZD8 CC in a 2:1 ratio. The switch I/II and interswitch regions of the GTP-loaded Rab7 form the binding interfaces, which correlates with the GTP-dependent interaction of PDZD8 and Rab7. Analysis of the protein interaction by isothermal titration calorimetry confirms that two Rab7 molecules bind the PDZD8 CC in a GTP-dependent manner. The structural model of the PDZD8 CC–Rab7 complex correlates with the recruitment of PDZD8 at the LE–ER interface and its role in lipid transport and regulation.

## Introduction

The lipid compositions of membranes are unique for each subcellular organelle in eukaryotes, and the proper distribution of lipids is crucial for the organellar function^1^. Most membrane lipids are synthesized in the endoplasmic reticulum (ER) and transported to other membranes by lipid trafficking pathways via vesicles or lipid transfer proteins (LTPs). Most organelles are connected with the ER network by forming membrane contact sites (MCSs), where the two membranes are closely apposed and tethered within a typical gap of 30 nm^2^. Non-vesicular lipid transport by soluble protein carriers at the MCSs plays a significant role in lipid distribution due to the efficiency and specificity of the lipid transport^2,3^.

The endocytic machinery sorts and degrades a variety of substances internalized by vesicles and vacuoles for intracellular homeostasis. The communication at the MCSs between the ER and late endosome (LE)/lysosome is critical for endosomal protein sorting, dynamics, and motility^4^. Endosomal maturation requires the sequential recruitment and activation of Rab GTPases, molecular switches that cycle between the inactive GDP- and active GTP-bound states. The GTP-loaded Rab7A (hereafter referred to as Rab7) on the LEs recruits various effectors facilitating endosome transport along microtubule tracks and their fusion with lysosomes and autophagosomes^5^. Rab7 recruits mainly through the switch regions, structurally diverse effector proteins such as PDZD8, FYCO1, RILP, and Rubicon^5^.

PDZD8 is an ER anchored lipid transfer protein containing a lipid-binding module of the synaptotagmin-like mitochondrial-lipid-binding (SMP) domain family^6^. PDZD8 consists of an N-terminal transmembrane helix, a SMP domain, a PDZ domain, a C1 domain, and the C-terminal coiled-coil domain. PDZD8 was known to form mitochondria-associated ER membranes (MAMs) and mediate the Ca^2+^ dynamics in neurons^6^. PDZD8 also localizes at the contact sites between the ER and late endosomes/lysosomes by interacting with the LE Rab7^7^. PDZD8 associates with an ER protein Protrudin and coordinates endocytic flow with lipid transport at the ER–endosome/lysosome interface^8–10^. The PDZD8–Rab7-Protrudin complex regulates the plus-end-directed movement of endosomes by recruiting kinesin-1, promoting the recycling and secretion of endosomes^11–13^. In contrast, the ORP1L–Rab7–RILP complex regulates the minus end-directed motility of endosomes by binding to the dynein motor complex^4^. The Rab-binding domains (RBDs) of PDZD8 and RILP bind only to the active form of Rab7^7,14^. The RBD of RILP adopts an α-helical conformation forming a symmetric coiled-coil dimer^14^. The RBD of at the C-terminal region of PDZD8 is predicted to have a coiled-coil (CC) structure of about 45 amino acids. However, due to the low sequence similarity to the proteins of known structures, the structure of the PDZD8 CC and its Rab7-binding mode were elusive.

In this study, to identify the structural determinants for the GTP-dependent interaction of PDZD8 and Rab7, we determined the structure of the human PDZD8 CC and Rab7-GTP complex. The PDZD8 CC forms a flat structure of three α-helices interacting with two Rab7 molecules on opposite sides of the coiled-coil. The PDZD8 CC associates with the active Rab7 by recognizing the switch I/II and interswitch regions. Our structural model of the PDZD8 CC–Rab7 complex is consistent with the role of PDZD8 in membrane tethering and lipid transport at the membrane contact sites.

## Results

### Overall structure of the PDZD8 CC–Rab7 complex

**Figure 1:**
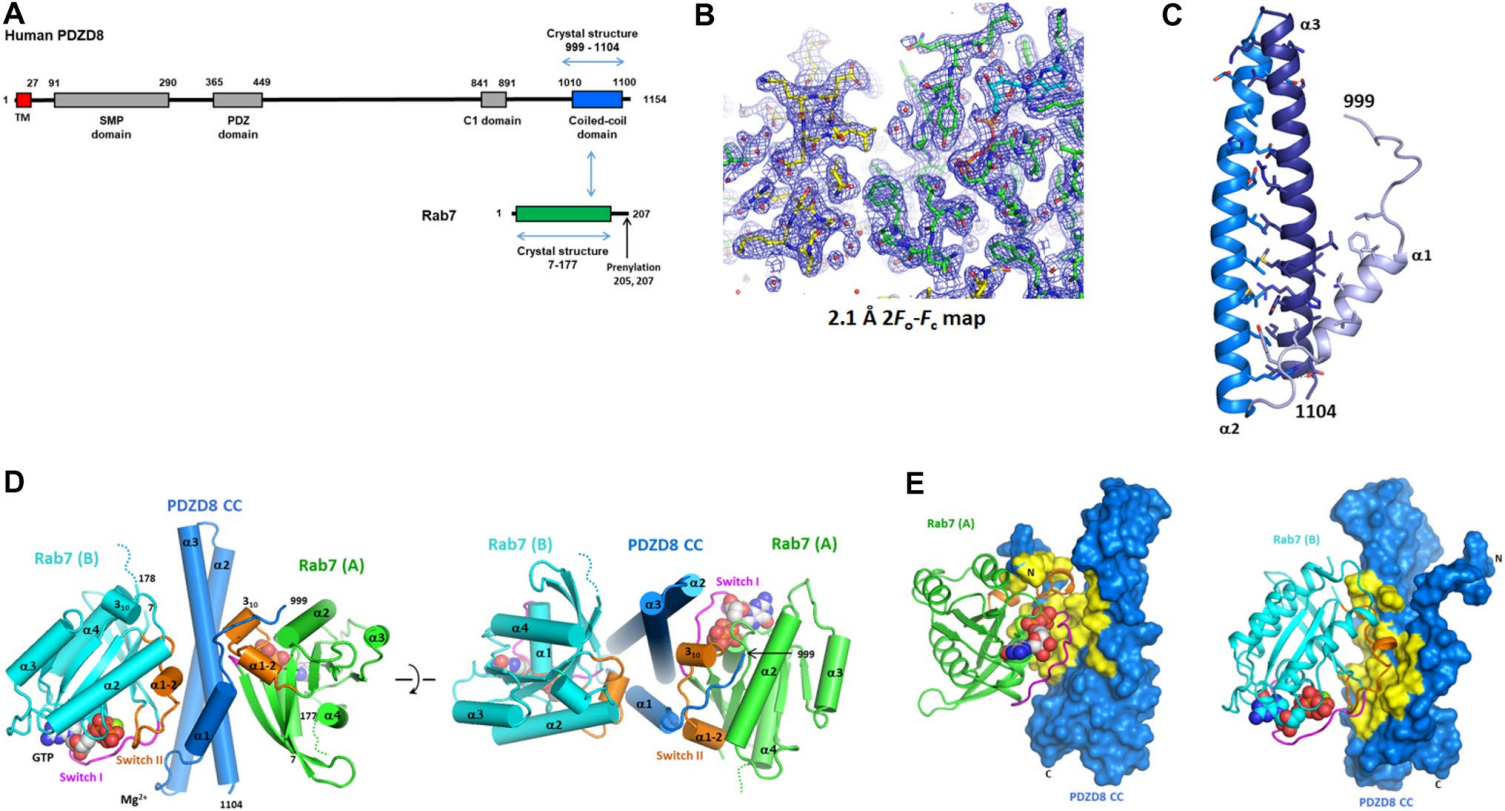
The overall structure of the human PDZD8 CC–Rab7 complex. (**A**) Schematic representation of the domain structures of human PDZD8 and Rab7. The domains with the structures determined in this study are indicated with blue arrows. (**B**) 2.1 Å 2*F*_0_–*F*_c_ electron density maps with the final model superimposed. The Rab7 structure is shown in green and the PDZD8 CC in yellow. (**C**) Overall structure of the PDZD8 coiled-coil domain. The residues in the interfaces between the helices are shown in stick models. (**D**) Cylindrical representation of the human PDZD8 CC–Rab7 complex. The bound GTPs and Mg^2+^ ions are shown in spheres. The disordered loops at the C-termini of Rab7 are indicated with dotted lines. The top view of the structure is shown in the right panel. The switch I residues were colored in magenta, and switch II residues in orange. (**E**) Binding interfaces of Rab7 and the PDZD8 CC. The PDZD8 CC is shown in surface representation with the residues interacting with Rab7 colored in yellow.

Human PDZD8 contains an N-terminal transmembrane helix (residues 2–27), followed by a lipid-binding SMP domain and a PDZ domain. The C-terminal region of PDZD8 contains a C1 domain (residue 841–891) and a coiled-coil domain (residue 1010–1100). The middle region (residue 449–841) connecting the PDZ and C1 domains is made of a long disordered loop of about 400 amino acids (Fig. 1A). To obtain a structural insight into the LE–ER tethering of PDZD8 by the PDZD8 CC–Rab7 interaction, we performed X-ray crystallographic studies of the PDZD8 CC domain in complex with the Rab7-GTP. The recombinant proteins of the PDZD8 CC and GTP-loaded Rab7 were purified individually. We tested the Rab7-binding activity of various PDZD8 CC constructs with different domain boundaries by isothermal titration calorimetry (ITC) and selected the smallest construct (residue 999–1104) for crystallographic studies. To obtain the crystals of the PDZD8 CC–Rab7 complex, the proteins were mixed in a 1:1 molar ratio and were incubated for one hour prior to crystallization setup. The hexagonal crystals of the PDZD8 CC–Rab7 complex with P6_5_ space group appeared in a week. The structure of the PDZD8 CC–Rab7 complex was determined at 2.1 Å resolution by molecular replacement method using a structure of the GTP-bound Rab7 (Table 1). In the asymmetric unit of the crystal, there were two molecules of Rab7 bound to a single molecule of the PDZD8 CC. The electron densities of GTP-ligands in the Rab7 molecules were clearly visible (Fig. 1B). The PDZD8 CC forms a coiled-coil structure within a single polypeptide chain and does not have intermolecular coiled-coil interaction. The PDZD8 CC starts with an N-terminal ten-residue loop followed by three α-helices (Fig. 1C). The first α-helix (residue 1010–1023) is short with 3.8-helical turns. The second and third α-helices form a 55 Å long antiparallel coiled-coil with ten helical turns. The three α-helices form a flat structure arranged in an α1-α3-α2 topology. The three antiparallel helices are held together mainly by hydrophobic interaction with additional three hydrogen-bonds and three salt bridges. The Rab7 molecules make an asymmetric bivalent binding to the single PDZD8 CC domain (Fig. 1D,E). Two Rab7 molecules (Rab7a-A and Rab7a-B) bind on the opposite sides of the flat CC structure and have no direct contact with each other. Both Rab7 binding sites in the PDZD8 CC contain hydrophobic patches on the surface.

**Table 1:** Data-collection and refinement statistics.

Crystal	PDZD8 CC–Rab7
Construct	PDZD8 CC 994–1123, Rab7 2–195
**Data collection**	
Beamline	PLS-7A
Wavelength (Å)	0.97950
Space group	*P*6_5_
Unit-cell parameters (Å)	*a* = 84.2, *b* = 84.2, *c* = 154.6
Resolution range (Å)	50–2.1 (2.14–2.10)
No. of reflections	397,776
No. of unique reflections	36,020 (1773)
Multiplicity	11.0 (11.3)
Mean *I*/*σ*(*I*)	53.9 (7.8)
Completeness (%)	99.7 (100.0)
*R*_merge_ (%)	8.7 (50.6)
Wilson *B* factor (Å^2^)	33.6
**Refinement**	
*R*_work_ (%)	20.7 (27.3)
*R*_free_ (%)	25.3 (34.9)
R.m.s.d., bond lengths (Å)	0.008
R.m.s.d., bond angles (°)	1.089
B factor (Å^2^)	
Overall	43.2
Molecule A, B (Rab7)	39.9, 46.5
Molecule C (PDZD8)	46.5
Ligand (GTP)	38.4
Water	43.5
No. of non-H atoms	
Protein	3589
Ligand	66
Solvent	169
**Ramachandran statistics**	
Favored (%)	96.9
Disallowed (%)	0.23
PDB entry	7F6J

### PDZD8 CC recognizes the conserved hydrophobic triad of Rab7 in the active conformation

**Figure 2:**
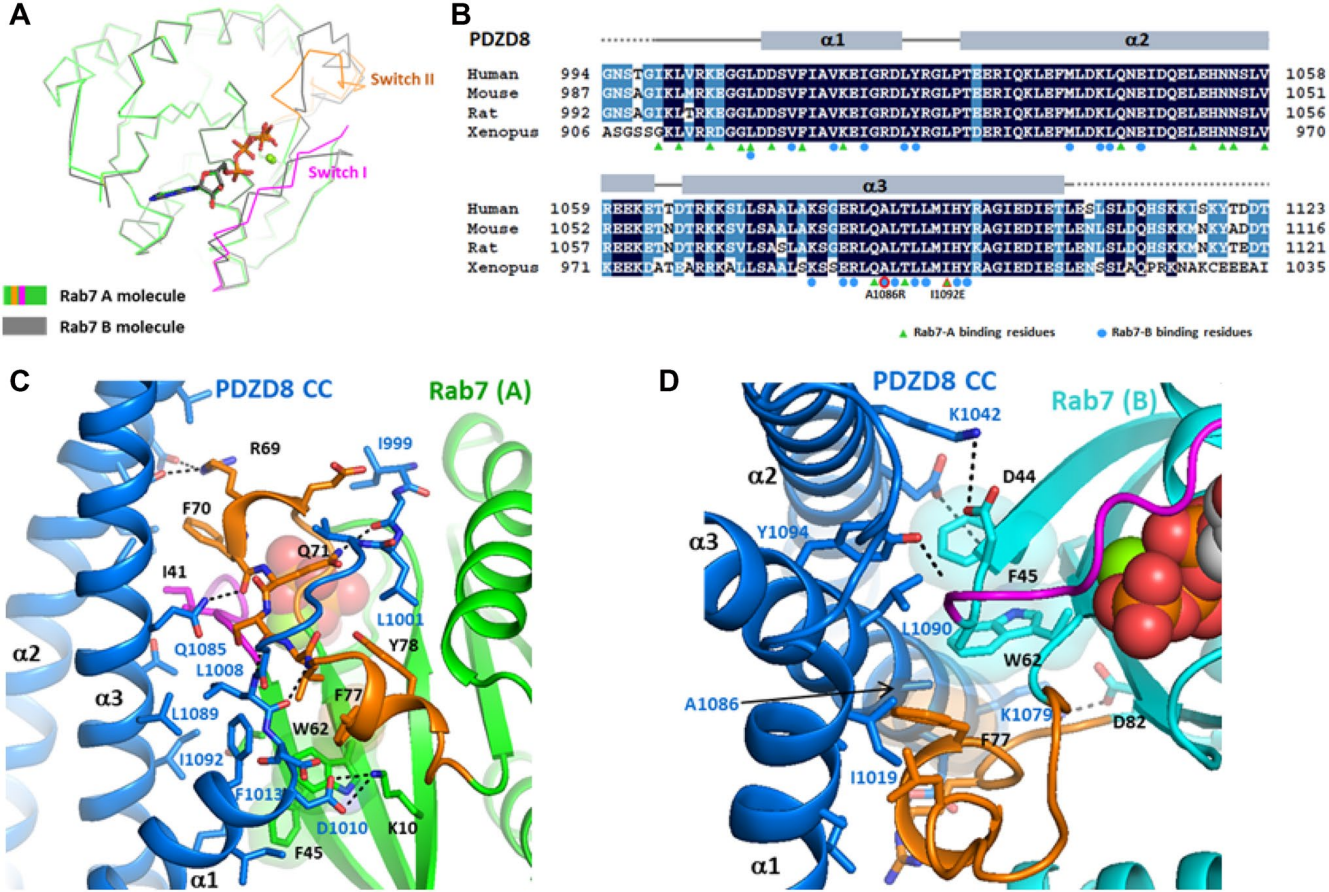
The binding interface of PDZD8 CC and Rab7. (**A**) Structural comparison of Rab7-A and Rab7-B. (**B**) The secondary structure elements of the PDZD8 CC domain. The PDZD8 residues contacting with Rab7 were indicated with green triangles for Rab7-A and blue circles for Rab7-B. The disordered residues in PDZD8 were indicated with dotted lines. The three PDZD8 residues mutated for the binding experiments were indicated with the red outlines. (**C**) The binding interface between the PDZD8 CC and Rab7-A. The residues forming the interface are shown in stick models. The hydrogen bonds are shown in dashed lines. The side chains of the hydrophobic triad (F25, W62, and F77) are indicated with transparent surfaces. (**D**) The binding interface between the PDZD8 CC and Rab7-B. GTP is shown in spheres.

The PDZD8 was known to bind Rab7 in a GTP-dependent manner^7,8,10^. The structure of the PDZD8 CC–Rab7 complex reveals the structural determinants of the GTP-dependent interaction. Two Rab7 molecules loaded with GTP have an active closed conformation of switch I and II loops. Rab7-A and Rab7-B have an almost identical structure except for slight conformational variation in the switch I and II loops with the C_α_ rmsd of 1.38 Å for 171 residues (Fig. 2A). Rab7-A forms two salt bridges and six hydrogen bonds with PDZD8. The PDZD8 CC utilizes four residues of the N-terminal loop and 11 residues of the three α-helices for Rab7-A binding, forming a buried surface area of 386 Å^2^ (Fig. 2B). The direct recognition of the PDZD8 CC on the closed conformation of switch loops explains the GTP-dependent interaction of PDZD8 and Rab7. The switch I and II loops of Rab7-A interact with the N-terminal loop and the helix α2/α3 of PDZD8. The N-terminal loop of the PDZD8 CC binds in the cleft between the helix α2 and the switch II loop, inserting L1001 into the hydrophobic pocket (Fig. 2C). Phe45 and Trp62 in the interswitch and Phe77 of switch II make hydrophobic contact with the helix α1. These three conserved aromatic residues adjacent to switch I and II regions were known as the hydrophobic triad, a major structural determinant for effector binding and specificity in Rab proteins^15^. In addition, Ile41 of switch I and Phe70 of switch II interact with the hydrophobic patch (Leu1051 and Leu1084) on the α2-α3 helices of PDZD8. Rab7-B contacts with the 19 residues of the PDZD8 CC, forming a buried surface area of 224 Å^2^ (Fig. 2B). The aromatic triad of Rab7-B forms a hydrophobic interaction with the flat surface on the α1, α2, and α3 helices of the PDZD8 CC (Fig. 2D). The α2 and α3 helices of the PDZD8 CC contact Ile41 and Gly42 of the switch I loop and the interswitch residues. The switch II loop of Rab7-B makes a hydrophobic contact with the shallow pocket between the α2 and α3 of the PDZD8 CC. Both Rab7-A and Rab7-B interact with PDZD8 using the switch I/II and interswitch regions as commonly observed in the Ras-like small GTPases in complex with their effectors^16^. Even though Rab7-A and Rab7-B bind the opposite sides of the PDZD8 CC, both Rab7 molecules utilize the conserved hydrophobic triad for PDZD8 binding, suggesting a conserved mode of Rab7 effector recognition.

### PDZD8 CC–Rab7 interaction is dependent on the GTP-binding state of Rab7

**Figure 3:**
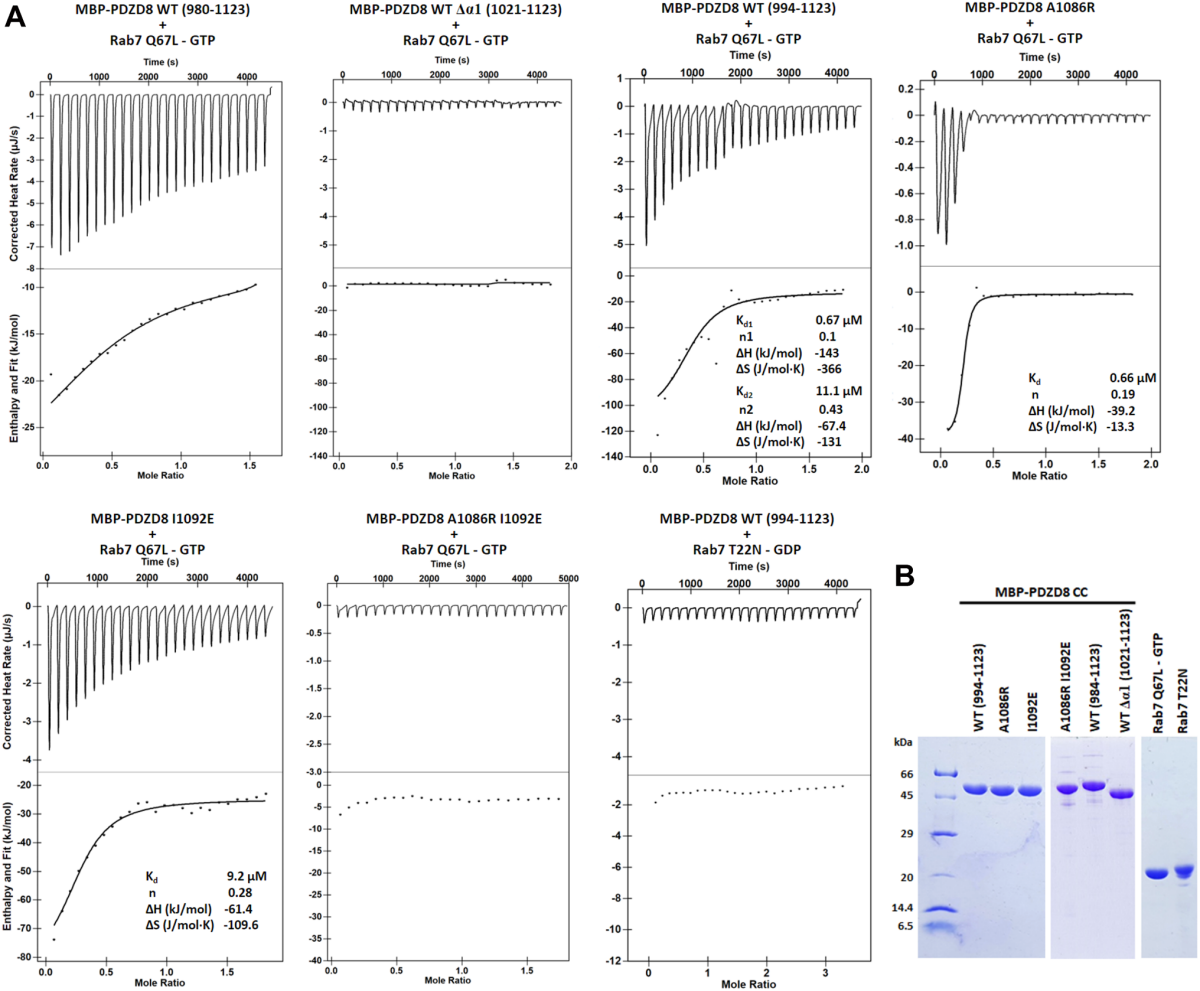
Affinity measurement of the PDZD8 CC and Rab7 interaction. (**A**) Measurement of binding affinity of the PDZD8 CC to Rab7 by isothermal titration calorimetry. Rab7 with a concentration of 0.1 mM was titrated with 1 mM of the MBP-PDZD8 CC constructs. The active Rab7 Q67L and inactive Rab7 T22N mutants were loaded with GTP and GDP, respectively. The ITC analysis was performed three times for each construct and a representative titration curve was shown for each construct. The other ITC results are shown in the supplementary information. (**B**) SDS-PAGE of the purified recombinant proteins used for the ITC experiments. The two gels were cropped from different parts of the same gel. The uncropped gel is shown in the supplementary information.

To analyze the GTP-dependency and stoichiometry of the interaction, we measured the affinity of the various PDZD8 CC and Rab7 constructs by ITC (Fig. 3A,B). To obtain homogeneous nucleotide-binding states of Rab7, we used the constitutively GTP-binding Rab7 Q67L and GDP-binding Rab7 T22N rather than wild-type Rab7 for ITC experiments. The PDZD8 CC constructs spanning residues 980–1123 and 994–1123 interacted with Rab7. However, the PDZD8 CC construct lacking helix α1 (1021–1123) did not bind Rab7. Since the crystal structure displayed the binding of two Rab7 molecules to a single PDZD8 CC molecule, we used a two-site binding model for the wild-type PDZD8 CC and Rab7 interaction. The MBP-PDZD8 CC bound two GTP-loaded Rab7 molecules with Kd values of 0.67 and 11.1 μM, respectively. The primary binding site displayed 16 times higher binding affinity than the secondary site. We interpreted that the high binding affinity represents the Rab7-A interaction considering the 1.7 times larger interface area of Rab7-A than the area of Rab7-B. To confirm the 2:1 binding mode observed in the crystal structure, we constructed two Rab7-binding site mutants of the PDZD8 CC. Ala1086 of PDZD8 is located in the center of the binding interface between PDZD8 and Rab7-B. Therefore, A1086R mutation was expected to interfere with the Rab7-B binding without affecting the interaction with Rab7-A (Fig. 2D). Ile1092 is located in the center of PDZD8–Rab7-A interface (Fig. 2C). The I1092E mutation was expected to disrupt the Rab7-A binding. As predicted from the structure analysis, PDZD8 A1086R retained the high-affinity binding with a Kd of 0.66 μM when analyzed by a single-site binding model. However, PDZD8 I1092E mutant displayed a Kd of 9.2 μM, interfering with the high-affinity binding of Rab7-A. Consistently, when both Rab7-A and Rab7-B binding sites were altered with A1086R/I1092E mutations, the affinity of PDZD8 to Rab7 was lost completely. These observations confirm that the PDZD8 CC has two Rab7 binding sites with different affinities and the two Rab7-binding sites are independent of each other. In addition, we tested the guanine nucleotide-dependency of Rab7-binding to PDZD8. The Rab7 T22N is a constitutive GDP-binding inactive mutant^17^. The PDZD8 CC wild type was unable to bind Rab7 T22N, confirming that PDZD8–Rab7 interaction is GTP-dependent. The ITC data is consistent with the structural features of the PDZD8 CC–Rab7 complex in that the switch I and II loops with active conformation serve as a binding surface for the PDZD8 CC. This observation is consistent with the previous reports that the ER/LE localization of PDZD8 is dependent on the GTP-bound Rab7^7,8^.

### Conservation of a binding mode in Rab7-effector complexes

**Figure 4:**
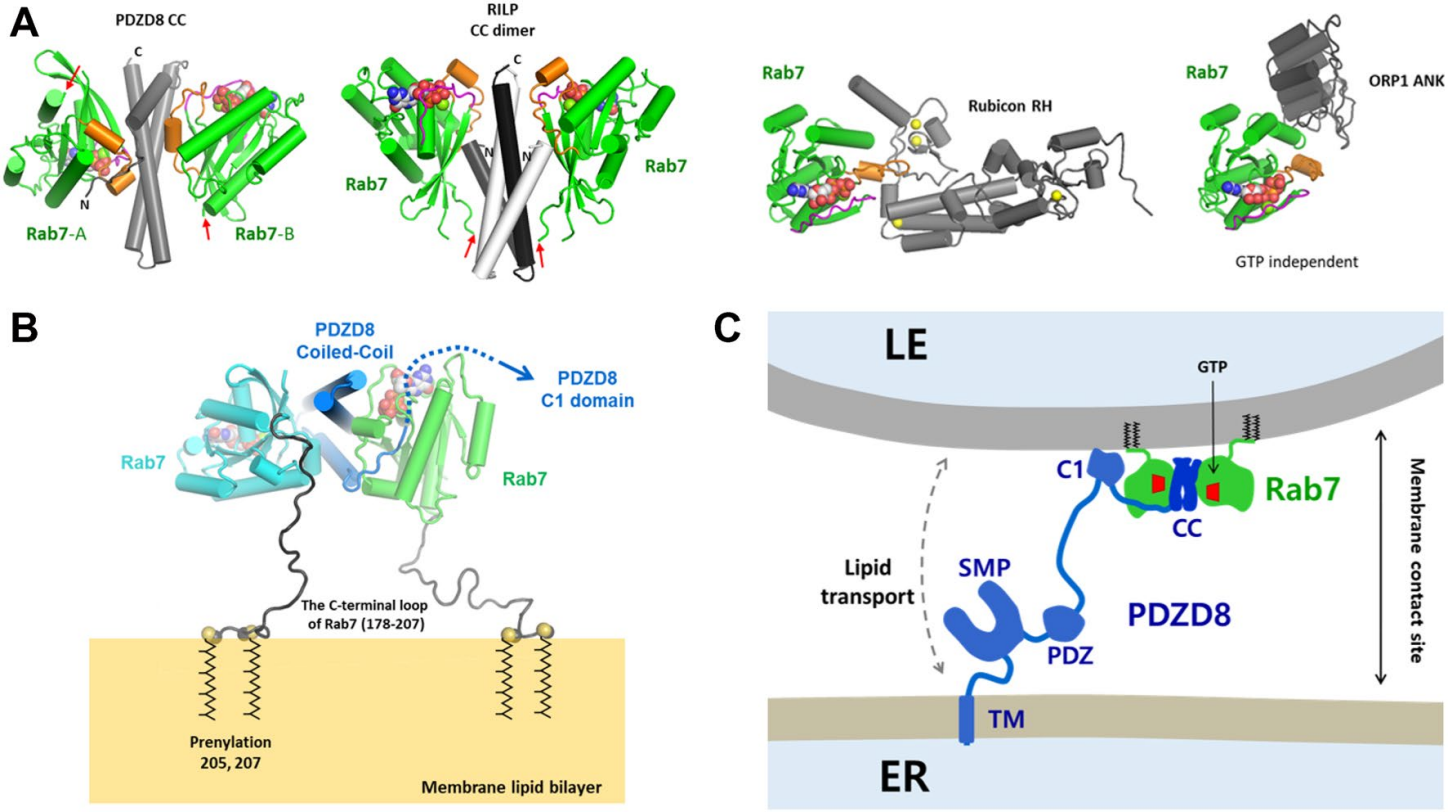
Schematic model of PDZD8 function at the LEL–ER membrane contact site. (**A**) Overall structures of Rab7 and its effector protein complexes. The structures of the PDZD8 CC–Rab7 (this study), RILP–Rab7 (PDB id: 1YHN)^14^, Rubicon RH–Rab7 (PDB id: 6WCW)^21^, and ORP1L–Rab7 (PDB id: 6IYB)^22^ were shown to compare the binding modes of the effector proteins. Rab7 and the effectors were colored in green and gray, respectively. The bound-GTP was shown in a sphere model. The red arrows indicate the C-termini of the Rab7 molecules. (**B**) Structural modeling of the PDZD8 CC–Rab7 complex on the lipid membrane. The C-terminal flexible loops (residues 178–195) of Rab7 invisible in the experimental structure were modeled with gray loops. The prenylation of the Rab7 residues at 205 and 207 was shown in thick lines. The upstream of the PDZD8 CC were indicated by blue dotted lines. The structure of the PDZD8 CC–Rab7 complex was oriented so that the C-terminal loop regions of two Rab7 molecules contact the lipid membrane at the same distance. (**C**) Schematic model of PDZD8 function at the LE–ER membrane contact site. The prenylated Rab7 localizes to the LE/lysosomal membrane. PDZD8 associates with two molecules of GTP-loaded Rab7 in the LE membrane by its coiled-coil domain. The PDZD8 contains an N-terminal transmembrane helix that anchors the protein to the ER and the C1 domain that binds PI(4)P and phosphatidylserine in the membranes^9^. The SMP domain of PDZD8 might transport various lipids between the membranes.

Structural comparison of the various Rab7-effector complexes suggests that the GTP-dependent interaction has a conserved mode of recognition on the active conformation of Rab7 (Fig. 4A). The hydrophobic triad on switch II and interswitch regions was first identified in Rab proteins and was proposed to be a major structural determinant for GTP-dependent effector binding and specificity^15^. So far, PDZD8^7^, RILP^18^, Rubicon^19^, and ORP1L^20^ were identified to be the Rab7 effector proteins with their structures reported. The PDZD8 CC, RILP CC, and Rubicon RH domains commonly recognize the conserved hydrophobic triad in the switch II and interswitch regions of Rab7^14,21^. However, the ORP1L ANK domain binds on the surface distal to the switch I/II regions, explaining the nucleotide-independent interaction of ORP1L^22,23^. The Rab7-binding domain of RILP is a coiled-coil homodimer and interacts with two Rab7-GTP molecules, forming a symmetric dyad complex^14^. The RILP–Rab7 complex exposes the C-termini of two Rab7 molecules in the same direction toward membranes. Although the PDZD8 CC does not form a coiled-coil dimer, two Rab7 molecules form a similar dyad configuration around the central PDZD8 CC monomer (Fig. 4A). Collectively, despite no structural and sequence similarities of the GTP-dependent Rab7 effector proteins, they share a common mode of Rab7 recognition.

### Structural model of the PDZD8 CC–Rab7 complex on the membrane

The structural model of the membrane-bound PDZD8 CC–Rab7 complex demonstrates the spatial arrangement of the subunits required for membrane recruitment (Fig. 4B). PDZD8 resides on the ER membrane by its N-terminal transmembrane domain and is targeted to the LEL membrane by its C-terminal coiled-coil domain via interaction with Rab7^7^. Rab7 is anchored to the LEL membrane via prenylation of the two cysteine residues in the C-terminus. Rab7 presumably lies far away due to their long flexible tails that precede the prenylation sites. The C-terminal cysteine residue is connected to the Rab7 core by a flexible 30 residue loop, which might allow the binding of two membrane-anchored Rab7 molecules to the PDZD8 CC in different orientations. We modeled a structure of the PDZD8 CC–Rab7 complex on the membrane considering the orientation of the Rab7 C-termini (Fig. 4B). The Rab7-binding domain of PDZD8 forms a flat coiled-coil structure, allowing binding of two Rab7 molecules simultaneously on the opposite sides of the PDZD8 CC. The asymmetric bivalent binding of Rab7 and the membrane anchoring by Rab7 impose a constraint in the orientation of the PDZD8 CC–Rab7 complex, positioning the coiled-coil domain parallel to the membrane surface (Fig. 4B). The switch I/II and interswitch regions of Rab7 contact with PDZD8, which makes the interaction GTP-dependent. The divalent binding of Rab7 effectors may help efficient tethering between the membrane contact sites by increasing the avidity of the interaction. The presence of the ER-targeting transmembrane helix and the Rab7-binding CC is consistent with the role of PDZD8 in membrane tethering and lipid transport at the membrane contact sites (Fig. 4C).

## Discussion

We investigated the structural basis of specific recognition of the GTP-loaded Rab7 by the PDZD8 CC. PDZD8 is an LTP tethered at the ER-endo/lysosome interface and contributes to endosome maturation in cooperation with Protrudin^9,10^. Recruitment of PDZD8 to the MCSs is mediated by the N-terminal TM region and the C-terminal Rab7-binding domain, which is crucial for its lipid transport function^7^. The bivalent Rab binding to effectors is common in many Rab GTPases^16^. The RBDs commonly adopt a coiled-coil dimer and bind to two Rab GTPases on the opposite sides of the central coiled-coil. Symmetric association of Rab GTPases to the coiled-coil dimer of the effectors were observed for the Rab5:Rabaptin5^24^, Rab6:GCC185^25^, Rab6:KIF20A^26^, Rab7:RILP^14^, Rab11:FIP2^27^ and Rab11:FIP3^28,29^ complexes. The bivalent Rab binding to effectors was suggested to increase the effector residence time at the membrane. It also imposes a restricted orientation to the effectors, allowing the effectors to project into the cytosol to perform their function^16^. Unlike the two-fold symmetry of typical Rab:effector complexes, a single molecule of the PDZD8 CC makes an asymmetric binding to two Rab7 molecules. The bivalent binding of PDZD8 to Rab7 seems to be advantageous in increasing the avidity of the interaction, which might provide stable tethering between the ER and LEs.

PDZD8 associates with an ER transmembrane protein Protrudin and regulates endosome trafficking through interaction with the GTP-bound form of Rab7^8,9,12^. The tripartite complex of PDZD8–Rab7-Protrudin is known to regulate the plus-end-directed movement of endosomes for the recycling and secretion of cargos. In contrast, the ORP1L–Rab7–RILP complex regulates the minus end-directed motility of endosomes for lysosomal degradation^16^. ORP1L is a cholesterol sensor/transporter which is recruited to the ER–LE/lysosome contact sites by association with Rab7^20,22^. Rab7 cannot bind PDZD8 and RILP/ORP1L simultaneously due to their overlapping binding sites, correlating with their role in the opposite directions of endosomal transport. Maturing endosomes undergo changes in the lipid composition, such as the depletion of phosphatidylserine and the enrichment of lysobisphosphatidic acid^30,31^. It implies that the direction of endosome sorting and endosome maturation might be controlled by associating with the specific Rab7-effectors including the lipid transfer proteins, PDZD8 and ORPL1.

In conclusion, this study demonstrates the key structural determinants for the GTP-dependent recruitment of PDZD8 to the LEs by Rab7-GTP. The bivalent Rab7-binding to PDZD8 might be advantageous for the stable tethering at the MCSs by providing avidities. PDZD8 recruited to the ER–LE interfaces by Rab7 might allow efficient lipid transport by its SMP domain for the late endosome/lysosome processing.

## Materials and methods

### Cloning of human PDZD8 CC and Rab7

The full-length clones for human PDZD8 (hMU002500, UniProt id: Q8NEN9) and Rab7 (hMU001574, UniProt id: P51149) were purchased from 21C Frontier Human Gene Bank (KRIBB, Daejeon, Republic of Korea). DNA encoding the C-terminal coiled-coil (CC) domain (residues 994–1123) of human PDZD8 was amplified by polymerase chain reaction and subcloned into the NcoI/XhoI site of a modified pHIS2 vector^32^. The PDZD8 CC was tagged with the N-terminal hexahistidines followed by a thrombin protease cleavage site (LVPR/GS). The DNA encoding human Rab7 (residue 2–195) was sub-cloned into the BamHI/XhoI site of the modified pHIS-2 vector. The dominant active GTP-binding (Q67L) and dominant-negative GDP-binding (T22N) mutants of Rab7 were prepared by point mutagenesis. *Escherichia coli* strain BL21(DE3) cells transformed with the plasmid encoding the PDZD8 CC or Rab7 were grown to an OD_600_ of 0.8 at 37 °C in LB medium. Cells were induced by adding isopropyl β-D-1-thiogalactopyranoside to a final concentration of 0.5 mM and were incubated for 12 h overnight at 20 °C prior to harvesting.

### Protein expression and purification

Cells expressing the PDZD8 CC were resuspended in 2X PBS buffer containing 20 mM imidazole (lysis buffer) and lysed by sonication. The supernatant containing the His-tagged PDZD8 CC was loaded to a Ni–NTA affinity column. The Ni–NTA column was thoroughly washed with the lysis buffer. The target protein was eluted from the column using a buffer containing 100 mM Tris–HCl pH 8.0 (final), 300 mM imidazole. The eluate was concentrated to 10 mg/ml using Amicon Ultra-15 centrifugal filter. The His-tag was cleaved by 10 international unit (IU) of thrombin protease (Reyon Pharmaceutical) per 10 mg of recombinant protein. The cleaved sample was subjected to size-exclusion chromatography on a HiLoad Superdex 200 column equilibrated with 20 mM Tris–HCl pH 8.0, 150 mM NaCl. The fractions containing the PDZD8 CC were concentrated by the centrifugal filter to 10 mg/ml for crystallization. The Rab7 Q67L or T22N mutant were purified by the same procedure as the PDZD8 CC. To inhibit GTP hydrolysis of the PDZD8–Rab7 complex required for crystallization, we used a dominant active Q67L mutant of Rab7. The purified Rab7 Q67L was supplemented with the final 1 mM of GTP.

### Crystallization and crystallographic analysis

To obtain the crystals of the PDZD8 CC–Rab7 complex, we mixed the purified PDZD8 CC and Rab7 proteins with a 1:1 molar ratio at a total 10 mg/ml, and incubated for 1 h at room temperature before crystallization setup. Preliminary crystallization experiments were carried out at 22 °C in 96-well crystallization plates using customized crystallization screening solutions by dispensing 0.8 μl protein solution and 0.8 μl precipitant solution. The hexagonal crystals of the PDZD8 CC–Rab7 appeared in a week using a solution consisting of 0.1 M Tris–HCl pH 8.0, 20% PEG 1000, 0.1 M NaCl in the 96 well plates. The crystallization condition was further optimized to 0.1 M Tris–HCl pH 8.0, 25% PEG 1000, 0.1 M NaCl using the hanging-drop technique in 15-well screw-cap plates. A drop consisting of 1.5 μl protein solution was mixed with 1.5 μl precipitant solution and equilibrated against 1 ml reservoir solution. High-quality crystals with dimensions of 0.1 × 0.1 × 0.15 mm appeared in a week. Crystals of the PDZD8 CC–Rab7 were cryoprotected in reservoir solution supplemented with 10% glycerol and flash-cooled by immersion in liquid nitrogen. Crystals were preserved in a cryogenic N_2_-gas stream (~ 100 K) during diffraction experiments. Native diffraction data for the PDZD8 CC–Rab7 were collected at a fixed wavelength of 0.97949 Å using an ADSC Q270 CCD detector on the 7A beamline at Pohang Light Source (PLS), Pohang Accelerator Laboratory. All data were processed and scaled using HKL-2000. The structure of PDZD8 CC–Rab7 complex was determined by molecular replacement using the structure of human Rab7 (PDB code: 1T91) as a search model. Two molecules of Rab7 were found in the asymmetric unit using the program Phaser^33^, and the density-modified map showed clear electron densities of the PDZD8 CC and Rab7. The final model including two molecules of Rab7 and one molecule of PDZD8 CC was refined to R_work_/R_free_ values of 21.1%/25.7% using the software Phenix^34^. The figures of all PDB structures were drawn using the software PyMOL (https://pymol.org).

### Isothermal titration calorimetry

The protein–protein interaction of the PDZD8 CC and Rab7 was analyzed by isothermal titration calorimetry (ITC) using an Affinity ITC calorimeter (low volume cell 190 μl; TA instruments) at 20 °C. The PDZD8 CC constructs used for the ITC experiments were fused to the His-tagged maltose-binding protein (MBP) in their N-termini for protein stability. The purified Rab7 Q67L and Rab7 T22N were loaded with 1 mM of GTP and GDP, respectively. All proteins were prepared in the identical buffer containing 20 mM Tris–HCl pH 7.5, 150 mM NaCl. The syringe was loaded with 1 mM of the MBP-PDZD8 CC and the cell was filled with 300 μl of 0.1 mM Rab7. The titration curve was obtained by injecting 2 μl × 25 aliquots of the MBP-PDZD8 CC into the cell at a time interval of 300 s. The enthalpy of reaction, ΔH0, the binding constant, K_d_, and the stoichiometry value, n, were calculated from the measured heat changes, δH_i_, upon the association of the MBP-PDZD8 CC and Rab7. The titration data were analyzed using the NanoAnalyze program (TA instruments) and fitted into a two-site binding model for wild type and an independent binding model for mutants.

## Supplementary Information

Supplementary Information.

## Data Availability

The coordinates and structure factors of the PDZD8 CC–Rab7 complex have been deposited in the Protein Data Bank with the accession code 7F6J.
