# Supplementary material for: Structural basis of human PDZD8–Rab7 interaction for the ER-late endosome tethering
Source: Sci Rep. 2021 Sep 22;11:18859. doi: 10.1038/s41598-021-98419-5 (PMC8458453; doi:10.1038/s41598-021-98419-5)
Supplement: Supplementary file 1 — Supplementary Information. [file 41598_2021_98419_MOESM1_ESM.pdf]

Supplementary Information

Structural basis of human PDZD8-Rab7 interaction for the ER-late endosome tethering

Haider Khan[1†], Lin Chen[1†], Lingchen Tan[1], and Young Jun Im[1*]

[1]College of Pharmacy, Chonnam National University, Gwangju, 61186, Republic of Korea

†These authors contributed equally to this work.

* Corresponding author

E-mail: imyoungjun@jnu.ac.kr (YJI)

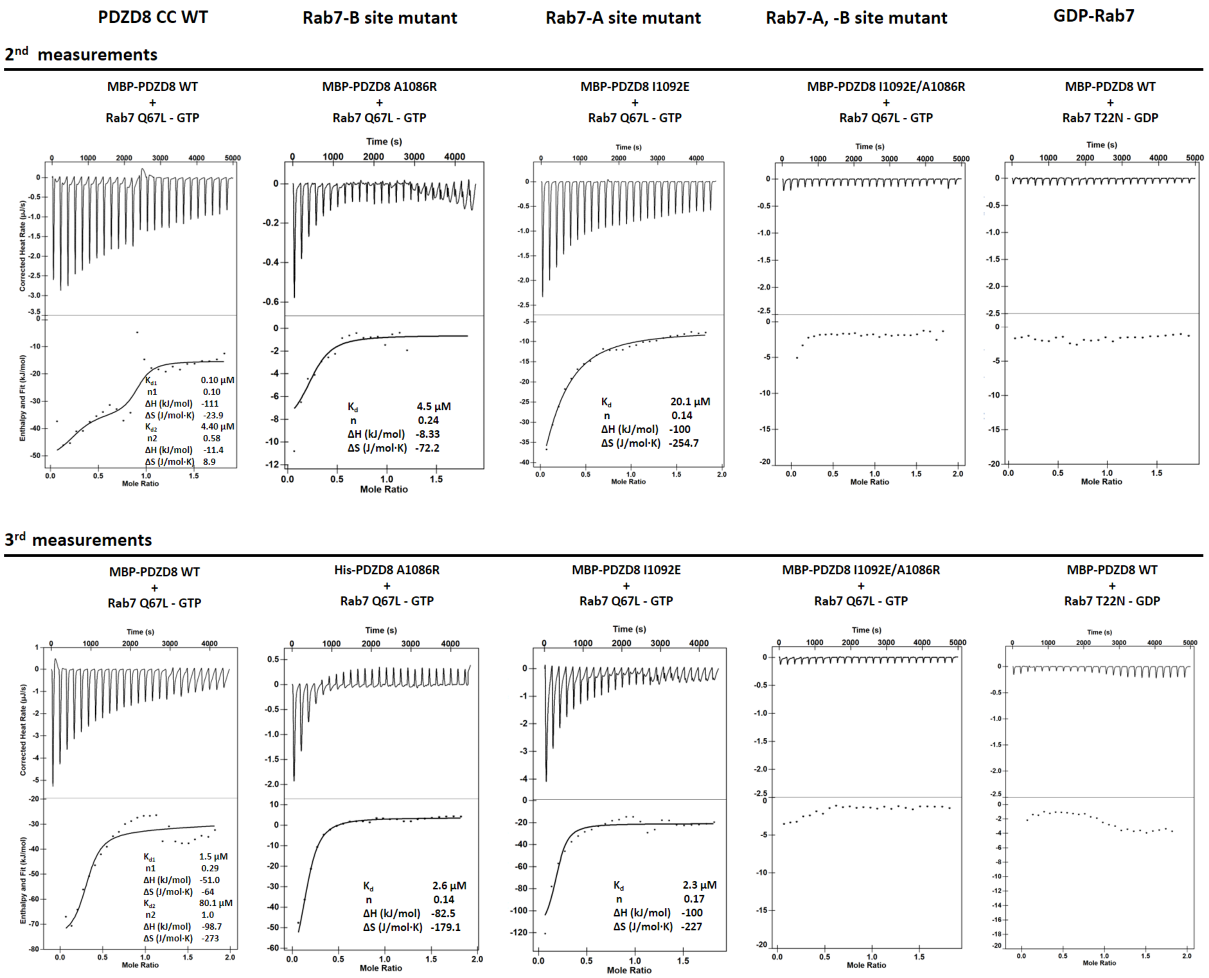

Figure 3A. Triplicated ITC results of each constructs. We have shown one representative ITC data for each construct in Figure 3A and the other two data sets in the supplementary information.

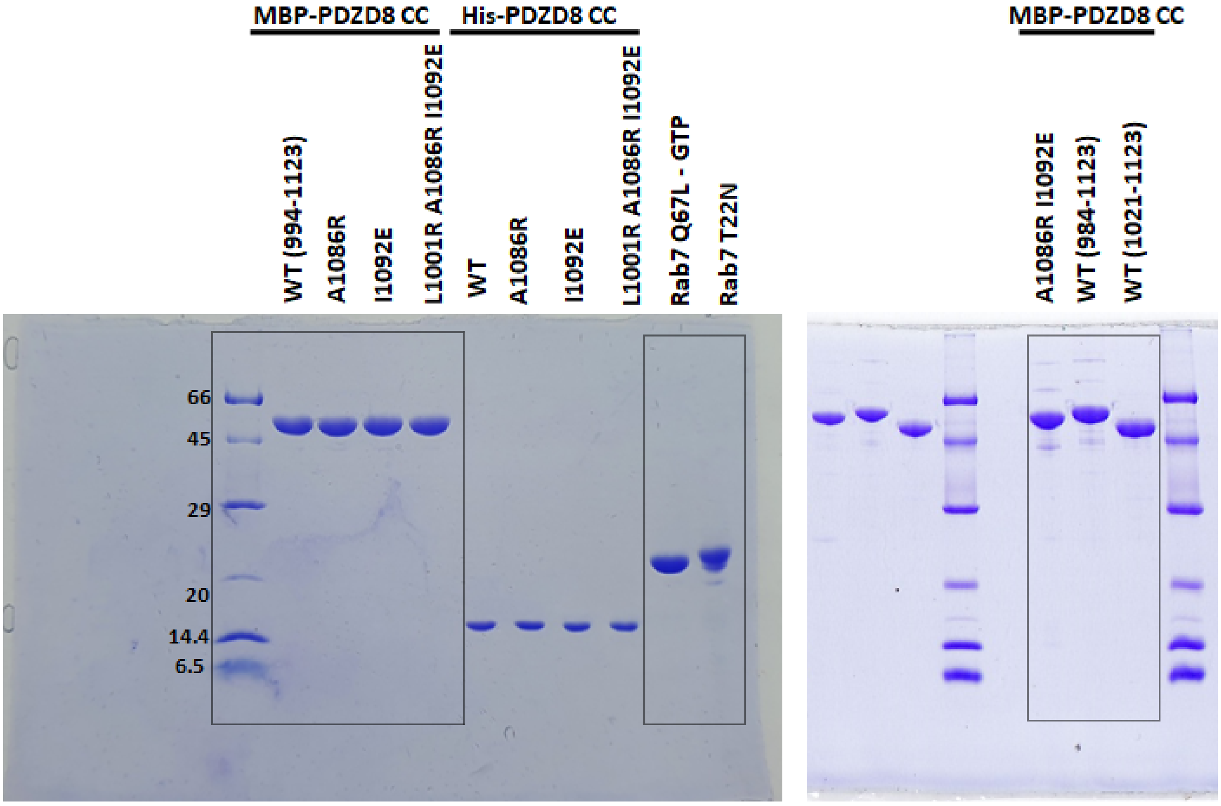

Figure 3B. An original SDS-PAGE image of the purified proteins used for ITC experiments. The cut images of the gels used for the figure 3B were indicated by dotted boxes.
